# A randomised controlled crossover study to assess adherence and palatability of a porridge supplement compared to a drink-based supplement in hospitalised older adults at risk of malnutrition

**DOI:** 10.1017/jns.2026.10108

**Published:** 2026-06-04

**Authors:** Samantha Jane Meredith, Kate A. Sheppard, Philip C. Calder, Harnish P. Patel, Stephen Eu Ruen Lim

**Affiliations:** 1 Faculty of Medicine, University of Southampton, UK; 2 NIHR Applied Research Collaboration Wessex, Southampton, UK; 3 University Hospital Southampton NHS Foundation Trust, UK; 4 NIHR Southampton Biomedical Research Centre, UK

**Keywords:** Appetite, Compliance, Energy intake, Oral nutritional supplement, Protein intake, Taste

## Abstract

Oral nutritional supplements (ONS) are essential adjuncts in managing malnutrition in hospitalised older people. This study aimed to explore the adherence and palatability of a new porridge supplement (PS) compared to a standard drink-based supplement (DS) in hospitalised older adults. A single-centre multi-method randomised controlled crossover study was conducted on adults aged ≥65 years at risk of malnutrition (Malnutrition Universal Screening Tool score 1–4). Each participant was prescribed PS (16 g protein; 249 kcal) and DS (18 g protein; 306 kcal) twice per day for 4 days in crossover sequence. ONS leftovers were weighed, and adherence calculated (% consumed). Palatability ratings were assessed with a 7-point Likert scale, and interviews were conducted with 9 older adults and 5 staff to explore product acceptability. Twenty-seven older people (mean age 80 years, 17 female) participated. Median daily PS intake (26.31% [IQR 10.23–48.02]) was significantly lower compared to DS (66.8% [IQR 26.29–75.37]), and texture of PS was significantly disliked compared to DS. There were no significant differences in the absolute volume consumed between products, and supplements did not replace normal dietary intake. Changes in physical ability and appetite, the hospital culture (e.g., staff attitudes), and palatability (e.g., thickness and taste) of products influenced ONS acceptability. To improve patient-centred nutritional care, participants requested a wider in-hospital range of food-based supplementation with natural ingredients and enhanced training for staff in nutritional care.

## Introduction

Globally around 19% of older people (aged ≥ 60 years) suffer malnutrition,^(1)^ defined as ‘a state resulting from lack of uptake or intake of nutrition leading to altered body composition (decreased fat free mass) and body cell mass leading to diminished physical and mental function and impaired clinical outcome from disease’.^(2)^ Prevalence of malnutrition differs across healthcare settings, with higher prevalence in residential care (17.5%), hospital (22–28%), and rehabilitation (29.4%) settings compared to older people living in the community (3.1–8%).^(3,4)^ Malnutrition increases hospital length of stay, escalates healthcare costs, is a key contributing factor in the aetiology of sarcopenia and frailty, and increases risk of morbidity and mortality.^(5–7)^ Considering the adverse clinical outcomes associated with malnutrition, the identification and management of malnutrition is a vital patient-centred outcome to enhance older adult’s health and quality of life and to enable cost-effective treatment and care.

Oral nutritional supplements (ONS) are essential adjuncts in managing malnutrition in hospitalised older people. ONS are energy- and nutrient-dense products designed to increase dietary intake when diet alone is insufficient to meet daily nutritional requirements.^(8)^ According to expert consensus, ONS should contain both micro and macronutrients and provide at least 400 kcal and a minimum of 30 g of protein per day to improve nutritional status and decrease risk of functional decline.^(9)^ Overall, research suggests favourable impacts of ONS on nutritional status and healthcare costs, while the impacts on functional outcomes and mortality are more controversial.^(10,11)^ Nevertheless, to effectively produce clinical benefits, patients need good adherence and acceptability to the products on offer in hospital.^(12–14)^

There are numerous factors that may influence ONS adherence including type, variety, volume, temperature, energy density, duration and timing of supplementation, presentation, and whether any instruction or assistance has been given.^(9)^ The European Society for Clinical Nutrition and Metabolism (ESPEN) recommend that adherence should be assessed regularly when ONS are offered to older people with malnutrition and the form of ONS should be adapted to suit patient’s taste and eating capacity.^(8)^ Higher energy density ONS appear to have better adherence rates than lower energy density ONS, possibly related to their smaller volume.^(12,15)^ Typically, research has investigated the adherence of ready-made multi-nutrient liquids in older patient groups and has found a mean adherence with ONS of 67% in hospital, 81% in the community and an overall adherence of 78.8% across healthcare settings.^(12)^ In other research, older hospitalised patient’s adherence with drink-based supplements was low (37%) and incurred a wasted cost of £18,294 per year across 4 geriatric wards in 1 hospital.^(14)^

Supplemental preference may be affected by a multitude of factors such as taste, colour, smell, aftertaste, and texture. Factors reducing drink-based supplement palatability and adherence included thicker textures,^(16)^ build-up of a mouthcoating,^(17)^ abdominal bloating,^(18)^ alterations in mouthfeel when wearing dentures, and changes in flavour perceptions depending upon medication status.^(19)^ Moreover, continual single use of a supplement resulted in monotony and taste fatigue leading to reduced intake.^(20)^ Typically, hospital patients are offered drink-based ONS. However, previous research has identified that 56% of older adults (*n* = 96, 58% female, 28% with malnutrition) in hospital did not like drink-based supplements.^(14)^ Hence, exploration of adherence to different ONS formats is an important research direction to maximise nutritional intake for malnourished older persons.

Hospitalised older adults with malnutrition should be offered an improved range and provision of ONS to suit personal preferences and maximise intake.^(9)^ Research suggests that alternative ONS products such as ice cream are acceptable by hospitalised older adults at risk of malnutrition.^(21)^ Yet this is an understudied area, with limited data investigating adherence to alternative ONS products compared to ready-made drinks in hospital, such as powdered ONS and snacks. ONS variations according to flavours and categories need to be considered to improve patients’ adherence. Therefore, this study aimed to investigate the adherence and palatability of a new porridge supplement compared to a standard drink-based supplement and to explore product acceptability in hospitalised older adults at risk of malnutrition.

The specific objectives included:

- To determine patient adherence to a new porridge supplement compared to standard drink-based ONS, assessed through measurement of product intake (% of product consumed).
- To examine the palatability of a new porridge supplement compared to standard drink-based ONS.
- To assess the acceptability of product use on acute medical wards through qualitative semi-structured interviews with participants and healthcare professionals.

## Methods

### Study design and setting

This was a single-centre multi-method randomised controlled crossover design. A crossover design was chosen to account for heterogeneity in an older patient population (e.g., differences in disease states and oral sensory perceptions), in which participants acted as their own controls to better compare palatability of products. This study was conducted at University Hospital Southampton (UHS) National Health Service (NHS) Foundation Trust on acute trauma and orthopaedic wards. The manuscript follows Consolidated Standards of Reporting Trials (CONSORT) guidance (Supplement 1).

### Ethics and data sharing

This study was conducted according to the guidelines laid down in the Declaration of Helsinki, and all procedures involving human participants were approved by the Health Research Authority (HRA) and Health and Care Research Wales (HCRW) on 6^th^ January 2023 (22/LO/0898). Written informed consent was obtained from all participants. The study was registered on ClinicalTrials.gov (NCT05620082) on 17^th^ November 2022, including access to the protocol and statistical analysis plan (https://clinicaltrials.gov/study/NCT05620082?cond=malnutrition&term=relish&rank=1). De-identified participant data is available on University of Southampton research data repository ‘Pure’.

### Patient and public involvement

A public and patient representative provided input into the study protocol, reviewed patient-facing materials, and was a part of the study management group. Before development of the new porridge supplement, palatability and taste of porridge was assessed by a group of older people hospitalised on medical wards at UHS. Flavour preferences were explored with a survey (*n* = 25; aged 86.23 ± 5.93 (mean ± standard deviation [SD]) years; 56% female). Analyses showed a median likability rating of 5 (IQR 3–6) ‘mildly like’ points for porridge. Participants flavour preferences were golden syrup, strawberry, and apple and cinnamon.

### Participant recruitment

Eligible participants on acute medical and orthogeriatric wards were identified by the clinical care team and approached by the research team to obtain informed consent. Inclusion criteria were adults aged 65 years or above, medium-high risk of malnutrition (Malnutrition Universal Screening Tool [MUST] score 1–4),^(22)^ and able to provide informed written consent. Exclusion criteria were a MUST > 4 (severely malnourished), body mass index (BMI) ≤ 15 kg/m^2^ requiring specialist dietetics input, chronic liver disease, renal failure (any stage), dysphagia, major surgery within the preceding month, a terminal illness, patients receiving end-of-life care, unable to eat by mouth (Nil By Mouth [NBM]), and requiring alternative ONS as advised by dietetic support. Initially, patients receiving ONS in the previous month were excluded; however, this impacted recruitment rates and was subsequently removed from the exclusion criteria on 31^st^ May 2024.

Eligibility criteria were developed in response to screening and recruitment trials by the study team, in collaboration with the dietetic team to ensure participant safety, and consistent with malnutrition screening policy and management at UHS. All patients admitted to UHS are assessed for risk of malnutrition within 24 hours using MUST.^(22)^ Patients with a medium (score 1), or high risk (score ≥ 2) of malnutrition receive nutritional support. If a patient has a medium MUST score, oral nutritional supplements can be offered as a part of their nutritional action plan.

Eligible healthcare professionals were approached for interview by the research team. Inclusion criteria were staff able to provide consent and working at UHS on the wards receiving the ONS intervention. A sub-sample of patients participating in the trial were invited for interview. Purposive sampling was used to include staff from a range of roles and a diverse group of patients, including age, sex, and adherence to the tested products.

### Sample size

A sample size of 50 was chosen based upon guidance from previous literature investigating adherence and palatability of ONS.^(12,17)^ In a systematic review of 49 studies assessing ONS adherence, studies included 1–4 groups with a median of 30 (interquartile range 23–41) participants in each group.^(12)^ Difficulties with recruitment resulted in changes to eligibility criteria, expanding the number of wards involved in the study and extending the study end date. Fifty participants was a target supported by study resources, achievable in the timeframe of the project, and consistent with lower recruitment rates obtained from previous research with older people in hospital.^(23)^

### Trial randomisation

Participants were randomised to groups by the lead researcher in a crossover procedure using a computer-generated randomisation tool, ‘Research Randomizer’,^(24)^ to minimise sequence effects and bias. Participants were allocated to one of two sequences at random in a 1:1 allocation to eliminate bias associated with group assignment while creating similar sized groups. Randomisation was performed before recruitment started, generating a list of participant ID numbers randomly assigned to each group. On recruitment to the study, researchers referred to the randomisation table to assign participants to groups.

### Adverse event reporting

Adverse events were reported to the chief investigator (CI) and principal investigator (PI) and recorded on the Case Record Form (CRF) stored in the study site file. All serious adverse events were reported to the study team (CI or PI) and then onto the study sponsor within 24 hours of the investigator becoming aware of the event. All study staff and clinicians in contact with participants were responsible for noting adverse events that were reported by the participant and making them known to appropriate medical staff. Participants were encouraged to contact their research nurse/team at the time of an event occurring.

### Intervention

Participants were prescribed ONS twice per day for 4 days, in addition to normal meals, in a crossover design. Two ONS products (Supplement 2) were tested for palatability and adherence including a new porridge supplement (treatment) and standard drink-based ONS (control). Each ONS product was tested over 2 days in a randomised crossover sequence. The products were offered twice per day, in-between breakfast and lunch and after dinner to reduce the detrimental long period of calorie absence experienced overnight.^(25)^ The porridge (treatment) was powdered and mixed with 170 ml boiling water in a pot using a measuring jug and included a choice of 5 flavours including golden syrup, apple and cinnamon, strawberries and cream, bananas and custard, and chocolate. The drink-based ONS (control) were ready-to-drink milkshakes. Participants were offered the ONS in a screw-topped plastic bottle and choice of a variety of flavours (e.g., vanilla, strawberry, chocolate, banana, mocha). ONS products were prepared and distributed to participants by health care professionals working on the wards.

### Data collection

#### Participant characteristics

Participant characteristics including age (years), domicile status, marital status, care provision, usual residence, reason for admission, frailty (Program of Research to Integrate Services for the Maintenance of Autonomy [PRISMA-7]),^(26)^ risk of sarcopenia (Strength, Assistance with walking, Rising from a chair, Climbing stairs, and Falls [SARC-F]),^(27)^ and appetite (Simplified Nutritional Appetite Questionnaire [SNAQ])^(28)^ were assessed at baseline. Participant’s C-reactive protein (CRP) (mg/L)^(29)^ and National Early Warning Score (NEWS)^(30)^ were recorded as markers of acute illness (recorded as part of standard care), a potential mediator of older adults’ appetite. Consistent with hospital site screening policy and nutrition management, the MUST tool was used to categorise participants at risk of malnutrition.^(22)^

Levels of CRP (mg/L) were measured on admission and used as an inflammatory marker with mild inflammation considered at levels 5–30 mg/L and above 30 mg/L indicating moderate to severe inflammation.^(29)^ Research suggests a CRP of 30 mg/L is a threshold of acute inflammation leading to reduced food intake.^(31)^ The NEWS system alerts the clinical care team to deteriorating adult patients in hospital through scoring routine bedside measures, such as oxygen saturation and pulse rate, leading to an overall NEWS risk score indicating a specific clinical response (Low: 1–4 aggregated score, Medium: score 5–6, High: score ≥ 7).^(32)^

SNAQ is a four-item tool comprising items 1, 2, 4 and 6 of the Council on Nutrition Appetite Questionnaire (CNAQ), assessing appetite, satiety, taste of food and number of meals per day respectively. SNAQ has a maximum score of 20, with a score of ≤14 indicating poor appetite.^(28)^ The SARC-F comprises five components: strength, assistance in walking, rise from a chair, climbing stairs and falls. The scores range from 0 to 10, with a score of equal to or greater than four being predictive of sarcopenia and poor outcome.^(27)^ The PRISMA-7 contains seven items with a positive score of three or more indicating frailty and has high accuracy in identifying frail older adults.^(26,33)^

#### Primary outcome measures

Primary outcome measures included adherence to ONS and palatability. Adherence to ONS was assessed via documentation of ONS leftovers at 2 timepoints, including after lunch (for ONS given in the morning) and early morning the following day (for ONS given after dinner). Leftovers were weighed (g) using weighing scales (Seca Model 875 digital weighing scale) and documented by the research team. Adherence was calculated as the percentage of the mean ONS consumed per day. Moreover, the mean intake of ONS energy (kcal/day) and protein (g/day) was calculated.

Palatability ratings, including appearance, smell, taste, sweetness, texture, thickness, aftertaste, mouth feel, and overall likability was assessed with a 7-point hedonic Likert scale (7 = definitely like, 6 = moderately like, 5 = mildly like, 4 = neither like nor dislike, 3 = mildly dislike, 2 = moderately dislike, 1 = definitely dislike). Hedonic scales have been used to assess palatability of ONS supplements across population groups^(15,21)^ and have good reliability.^(34)^ Palatability ratings (score 1–7) were completed on initial introduction of each ONS, on day 1 and day 3.

#### Secondary outcome measures

Secondary outcome measures included acceptability of ONS products, total caloric intake (kcal/day), and the gap (%) in energy and protein intake compared to personal requirements. Existing literature suggests that the effectiveness of ONS in improving energy intake can be compromised by partial displacement of normal meals.^(35)^ Therefore, total energy intake was assessed through completion of patient food charts, including estimated proportion of meals consumed. Food charts were a part of standard care and were completed by the nursing team. Energy intake (kcal) was calculated from diaries via nutritional data supplied by hospital catering (Serco). The food diary was completed 2 days before the start of testing to estimate normal total energy intake and then completed daily during the 4-day testing period.

Acceptability of ONS products was explored with semi-structured interviews. The interviews explored the views of hospitalised older adults and healthcare professionals, exploring the acceptability of the ONS products and the factors influencing implementation (Supplement 3). The interviews were audio-recorded for data collection purposes and took place one-to-one with the researcher (SJM) within UHS in-person, or remotely via telephone, or Microsoft Teams. Patients were interviewed following completion of 4 days of ONS intervention.

#### Data analysis

Statistical analysis was conducted using the statistical software SPSS (V.29). Descriptive statistics (median (interquartile range [IQR]); mean (standard deviation [SD]); number (%)) were used to analyse ONS ingested (%), intake of energy (kcal/day), intake of protein (g/day), gaps to dietary requirements (%), and palatability Likert scores (score 1–7). Normality tests, including skewness and kurtosis, were conducted to determine suitable statistical analyses for measures (i.e., non-parametric vs. parametric tests). Primary outcome measures were compared between groups (treatment vs. control) using a related-samples Wilcoxon signed-rank test with asymptomatic significance (2-sided test), and differences in ONS intake (g) were compared between morning and evening supplements. Total daily energy intake (kcal) was compared between ONS groups and control (i.e., before testing) with a related-samples Friedman’s Two-Way Analysis of Variance (ANOVA) by Ranks summary to assess the impact of ONS on normal meal intake, using adjusted significance for pairwise comparisons. Participant’s energy and protein requirements were calculated using European guidelines recommending older people consume 1 g/kg of body weight protein and 30 kcal/kg of body weight energy daily.^(8)^ Participants absolute gap in energy (kcal) and protein (g) intake compared to their requirements was calculated and converted into a percentage before comparing between timepoints (baseline, porridge and drink-based) with a general linear model repeated measures ANOVA. Mauchly’s test of sphericity was used and Greenhouse-Geisser correction applied for data where sphericity was not assumed. Pairwise deletion was used to handle missing data.

Interviews were transcribed verbatim and analysed using reflexive thematic analysis (RTA).^(36)^ RTA is a method for identifying, analysing, and reporting patterns or themes within data. Organisation of qualitative data was conducted using NVIVO. Transcribed text was read and coded by SJM. The codes were analysed to generate concepts and ideas to determine the acceptability of ONS products and to identify facilitators and barriers to their implementation. The codes acted as tags or labels to help catalogue key concepts embedded within the raw data. From the codes, themes were developed to reflect the views and experiences of the patients and healthcare professionals regarding the intake and use of ONS products in hospital. Using an iterative approach, SERL acted as a ‘critical friend’, helping SJM to reflect and sense-make, interpret the barriers and facilitators to supplement adherence, and support the lead author as she reflected on her beliefs and assumptions.

## Results

**Figure 1.**
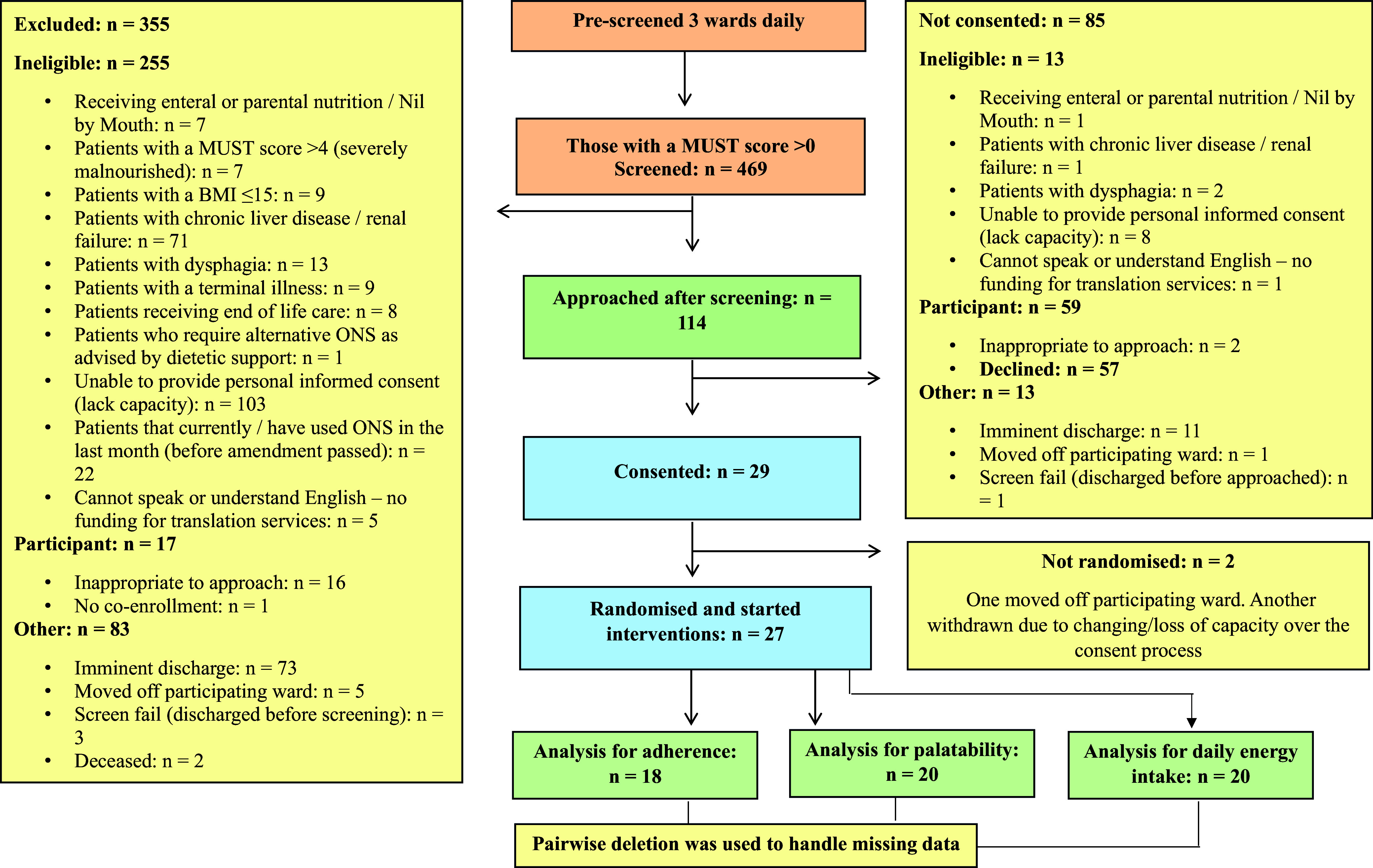
Flow diagram illustrating participant screening, recruitment, and analysis. Recruitment took place 29/04/24–31/12/24.

Four hundred and sixty-nine participants were screened, 114 participants were approached, and 27 consented to participate in the study (Figure 1). Participant characteristics are presented in Table 1. Participants were mainly female (63%), had a mean age of 80 years, had poor appetite (52%), were frail (67%), and were at risk of sarcopenia (74%). Based on MUST scores, 74.1 % of participants were at high risk of malnutrition. No adverse events were caused by the nutritional products studied.

**Table 1.** Participant characteristics

Characteristics	Participants (*n* = 27)
Age^a^ (years)	80.9 ± 8.6
	60–100 (range)
Sex	
Male	10 (37%)
Female	17 (63%)
Ethnicity	
White British	26 (96.3%)
Any other White background	1 (3.7%)
Marital status	
Divorced	5 (18.5%)
Married	13 (48.1%)
Widowed	9 (33.3%)
Care	
No care	21 (77.8%)
Formal provision	3 (11.1%)
Informal provision	3 (11.1%)
Residence	
Private home living alone	11 (40.7%)
Private home living with family	13 (48.1%)
Sheltered accommodation	3 (11.1%)
MUST score	
Medium risk (score of 1)	7 (25.9%)
High risk (score ≥ 2)	20 (74.1%)
PRISMA-7 frailty classification^b^	3 (2-4)
Frail	18 (66.7%)
Not frail	9 (33.3%)
SARC-F classification^b^	6 (3–8)
Sarcopenia	20 (74.1%)
No sarcopenia	7 (25.9%)
Short nutritional appetite questionnaire^b^	13 (11–15)
Poor appetite, high risk malnutrition	14 (51.9%)
Good appetite, low risk malnutrition	13 (48.1%)
Number of medications^b^	4 (3–7)
	0–20 (range)
C-Reactive protein (mg/L)^b^	47 (10-87)
Threshold for reduced food intake (≥30mg/L)	16 (59.3%)
National early warning score^b^ low risk	1 (0–2)
	27 (100%)
Height (cm)^a^	164.89 ± 10
Weight (kg)^a^	64.71 ± 11.95
Personal protein requirement (g/day)^a^	64.71 ± 11.95 44.85–86.80 (range)
Personal energy requirement (kcal/day)^a^	1941.37 ± 358.55 1345.50–2604 (range)
BMI (kg⋅m^2^)^b^	23.1 (19.8–28.3)
Underweight (<23)	13 (48.2%)
Healthy weight (23-29.9)	8 (29.6%)
Overweight (>29.9)	6 (22.2%)
Reason for admission	
Fracture	19 (70.4%)
Heart failure	2 (7.4%)
Pneumonia	2 (7.4%)
Subarachnoid haemorrhage	1 (3.7%)
Cellulitis	1 (3.7%)
Cancer	1 (3.7%)
Subdural haematoma	1 (3.7%)

*Note:* a, mean ± standard deviation; b, median and interquartile range; MMSE, mini-mental state examination; MUST, Malnutrition Universal Screening Tool; BMI, body mass index. Personal protein and energy requirements were calculated based on ESPEN guideline on clinical nutrition and hydration in geriatrics, comprising 1 g/kg of body weight for daily protein intake and 30 kcal/kg of body weight for daily energy intake.

### Adherence

There were no significant differences in the absolute volume of product consumed between the porridge (median 111.6 g [IQR 43.4–203.7]) and drink supplement (median 167 g [IQR 65.73–188.43]) (*p* = 0.981; *r* = 0.003). However, the daily percentage of porridge consumed (median 26.31% [IQR 10.23–48.02]) and energy (kcal/day) and protein (g/day) intake were significantly lower compared to the drink supplement (median 66.8% [IQR 26.29–75.37]) (Table 2). There were no differences in porridge intake (g) between morning (median 57.05 [IQR 14.44–108.59]) and evening (median 42.98 [IQR 0–100.9]) supplements (*Z* = −0.60; *p* = 0.546), and there were no differences in drink-based intake (g) between morning (median 90.95 [IQR 37.08–107.78] and evening (median 92.55 [IQR 50.98–105.3]) supplements (*Z* = −0.17; *p* = 0.862).

**Table 2.** Adherence to porridge and drink-based supplements (*n* = 18). Values are presented as median and interquartile range

	Porridge supplement (212.10 g)			Drink-based supplement (125 g)			
	Morning	Evening	Total daily intake*	Morning	Evening	Total daily intake*	*Statistic; level of significance; effect size
**Intake (g)**	57.33 (23.55–115.53)	42.98 (0–100.9)	111.6 (43.4–203.7)	89.35 (22.21–106.98)	87.23 (41.9–97.75)	167 (65.73–188.43)	*Z* = −0.02; *p* = 0.981; *r* = 0.003
**Proportion (%)**	27.03 (11.11–54.47)	20.26 (0–47.57)	26.31 (10.23–48.02)	71.48 (17.77–85.58)	69.78 (33.52–78.2)	66.8 (26.29–75.37)	*Z* = −2.49; *p* = 0.013; *r* = 0.42
**Energy (kcal)**	78.48 (40.45–142.6)	55.01 (0–126.48)	141.41 (63.13–249.32)	236.72 (74.71–270.97)	232.59 (120.85–253.04)	450.69 (198.69–492.33)	*Z* = −3.05; *p* = 0.002; *r* = 0.51
**Protein (g)**	5.04 2.6–9.16)	3.54 (0–8.13)	9.09 (4.06–16.02)	13.93 (4.4–15.94)	13.68 (7.11–14.89)	26.51 (11.69–28.96)	*Z* = −2.96; *p* = 0.003; *r* = 0.49

### Palatability

Participants found the drink-based ONS more palatable than the porridge (Table 3). Specifically, the texture of the porridge (median score 4 [IQR 3–7]) was significantly disliked compared to the drink-based control (median score 6 [IQR 4–7]) (*Z* = 2.39; *p* = 0.017; *r* = 0.38). Though not significant, 71% of participants agreed they liked the drink-based ONS and would be happy to choose it in future, compared to 45% for the porridge (*Z* = 1.86; *p* = 0.063; *r* = 0.28).

**Table 3.** Palatability of fortified porridge and drink-based oral nutritional supplements (values are expressed as median and interquartile range)

	Fortified porridge	Drink-based	Statistic; level of significance; effect size
**Appearance** **(*n* = 20)**	5 (4–6)	6.5 (4–7)	*Z* = 1.66; *p* = 0.096; *r* = 0.25
**Smell** **(*n* = 20)**	4 (4–5.25)	5.5 (4–7)	*Z* = 1.63; *p* = 0.104; *r* = 0.25
**Taste** **(*n* = 20)**	4 (2–6.25)	6.5 (4.25–7)	*Z* = 1.73; *p* = 0.083; *r* = 0.26
**Sweetness** **(*n* = 20)**	4 (3–7)	4.5 (2.25–7)	*Z* = 0.38; *p* = 0.704; *r* = 0.06
**Texture** **(*n* = 20)**	4 (2–6)	6 (4–7)	*Z* = 2.39; *p* = 0.017; *r* = 0.36
**Thickness** **(*n* = 20)**	5 (2.75–7)	6 (4–7)	*Z* = 0.75; *p* = 0.454; *r* = 0.11
**Aftertaste** **(*n* = 20)**	4 (2.75–5)	4 (2.25–7)	*Z* = 1.14; *p* = 0.253; *r* = 0.17
**Feeling in mouth** **(*n* = 19)**	4 (1.5–5)	4 (3–7)	*Z* = 0.76; *p* = 0.446; *r* = 0.12
Future choice* **(*n* = 20)**	4 (1.75–6)	6 (4–7)	*Z* = 1.86; *p* = 0.063; *r* = 0.28

*Note:* 7, definitely like; 6, moderately like; 5, mildly like; 4, neither like nor dislike; 3, mildly dislike; 2, moderately dislike; 1, definitely dislike. *7, strongly agree; 6, largely agree; 5, agree somewhat; 4, neither agree nor disagree; 3, disagree somewhat; 2, largely disagree; 1, strongly disagree.

### Daily energy and protein intake

Friedman’s Two-Way ANOVA showed a significant difference in daily energy (kcal) (Chi^2^ = 11.1; *p* = 0.004) and protein (g) intake (Chi^2^ = 7.92; *p* = 0.019) between baseline, porridge, and drink-based timepoints (excluding ONS intake). Pairwise comparisons using adjusted significance indicated a significantly higher energy (kcal) and protein (g) intake at the porridge timepoint compared to baseline (*p* = 0.003 and *p* = 0.017, respectively). Therefore, supplements did not reduce normal dietary intake (Table 4).

**Table 4.** Daily energy (kcal) and protein (g) intake at baseline, at porridge timepoint, and drink-based supplementation timepoint excluding and including supplements, and the percentage gap to personal nutrition requirements

	Baseline	Porridge supplement timepoint	Drink-based supplement timepoint	Statistic; level of significance (*n* = 20)
**Energy (kcal)**				
**Excluding supplement^a^**	569.75 (410.13–804.75)	718 (593–979.5)	774.69 (580.31–931.25)	Chi2 = 11.1; *p* = 0.004
**Including supplement^b^**	601.72 ± 303.52	1075.67 ± 411.20	1087.96 ± 311.13	F _(2,32)_ = 17.54; *p* = < 0.001
**Gap to requirement (%)^b^**	68.81 ± 16.97	44.52 ± 19.56	44.19 ± 17.16	F _(2,32)_ = 10.97; *p* < 0.001
**Protein (g)**				
**Excluding supplement^a^**	24.58 (17.45–35.21)	32.02 (21.95–47.28)	35.06 (21.22–40.66)	Chi2 = 7.92; *p* = 0.019
**Including supplement^b^**	28.27 ± 18.69	47.93 ± 18.56	52.34 ± 18.25	F _(2,32)_ = 12.47; *p* = < 0.001
**Gap to requirement (%)^b^**	59.81 ± 16.81	26.47 ± 26.72	20.52 ± 21.51	F _(2,32)_ = 18.40; *p* < 0.001

*Note:* a, median and interquartile range; b, mean ± standard deviation. Baseline data were collected 5.56 ± 4.34 (mean ± SD) days after patient admission.

There was a significant difference in the gap (%) between total daily energy and protein intake (including supplements) and personal energy and protein requirements between timepoints (*p* < 0.001). Pairwise comparisons showed significant differences in gaps to energy and protein requirements (%) between baseline and porridge timepoints (*p* = 0.002, *p* < 0.001, respectively) and between baseline and drink-based supplement timepoints (*p* < 0.001), however, there were no significant differences in the gap (%) to energy and protein requirements between porridge and drink-based supplement timepoints (*p* = 0.958, *p* = 0.401, respectively) (Table 4).

### Acceptability of nutritional supplements

**Figure 2.**
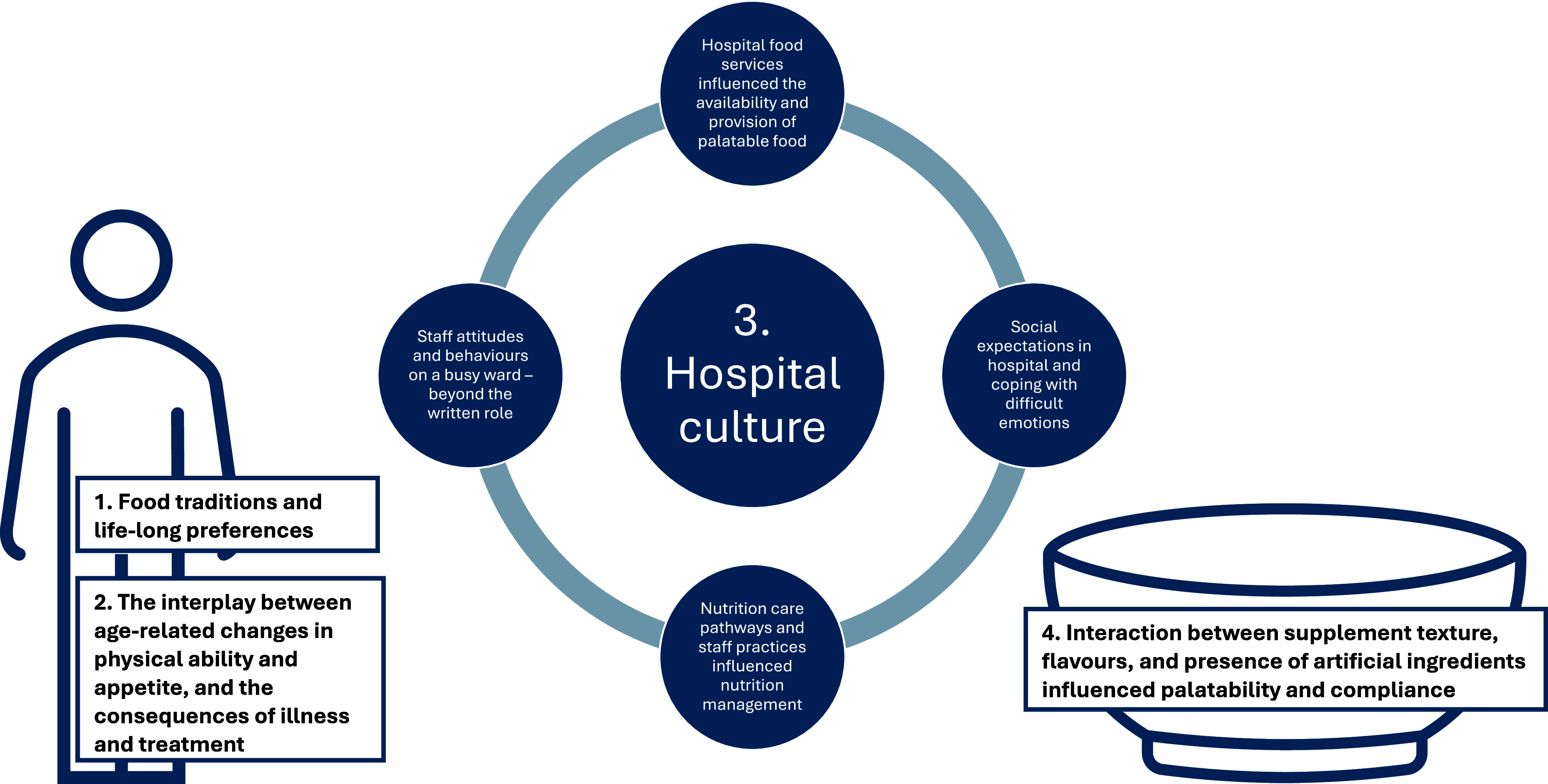
Main themes showing the factors influencing food intake, supplement palatability, and compliance.

Nine older adults (aged 65–95 years; 7 female), 2 nurses, 1 health care assistant, 1 dietetic assistant, and 1 dietitian were interviewed. Mean interview length was 22.64 minutes. Four main themes showcased the factors influencing food intake and acceptability of supplements, illustrated in Figure 2, and described below. Supporting quotations for each theme are presented in Table 5.

**Table 5.** The main themes and subthemes influencing the acceptability of nutritional supplements have been displayed with supporting quotations

Main theme	Quote examples	
Food traditions and lifelong preferences	‘Well, I’m not a very big eater. I’m a salad sort of person. I don’t eat a huge meal, not masses…. I like to have a small meal, but I think it’s fairly nutritious. We usually have a meat and two veg; maybe a sweet afterwards.’ (Participant 6) ‘We’re not people in this house to have low calorie yoghurts or anything like that. And my husband likes full milk. We have normal full cream yoghurts, really for him cos it’s better cos he’s coeliac. But I think there’s more value in them. We have real butter, but we don’t use a lot of it. I’d rather have that, and less of it, than margarine… Don’t like processed (food) very much.’ (Participant 7) ‘What we see with the older people they, because they grew up, and they’re so used to that sort of standard three meals a day with nothing in between. They’re not big on snacking…. So, trying to get them to have things between meals is something so outside of their normal that they struggle with it.’ (Dietetic assistant)	
The interplay between age-related changes in physical ability and appetite, and the consequences of illness and treatment	‘When you don’t feel like eating, something just to drink is easier than having something solid…. I don’t eat huge meals. And if I get a huge plateful looking at me, I think it puts me off.’ (Participant 13) ‘I’m a very slow eater. I seem to have to chew the food more thoroughly. And because I’m so slow it gets cold, and then I don’t enjoy it much. I like fish; we eat quite a lot of fish.’ (Participant 22) ‘Most of them forget their dentures. So, a lot of the elderly would have worn dentures. So, if they don’t plan to come in (hospital), I think, with a trauma or fracture…. they haven’t brought their dentures with them. So, it’s making sure that they are being given soft options if appropriate.’ (Dietician) ‘I think they need a bit more taste in their (food). So, because our patients, are not well and your taste bud is not good, especially when you’re having medication.’ (Health care assistant)	

Main theme	Subtheme L1	Quote examples
Hospital culture impacted food intake	Hospital food services influenced the availability and provision of palatable food	‘The meals weren’t bad, so I can’t really complain… You hear horror stories about hospital food, but I was quite happy with it… I didn’t eat a lot; I’ve lost a stone while I was in there. But I usually had cereal for breakfast or toast; lunchtime I usually had a sandwich, I usually only had half of it. But they were good, it was fine. And then in the evening at first, I wasn’t having much, just a sandwich, but then I started having the meals. Some were quite good; I was quite pleased with them.’ (Participant 25) ‘I didn’t think they were very sensitive if you were just coming out of an operation. They said, ‘what sandwich would you like?’ Well, if you’ve come through an operation like that, you don’t really want a sandwich. And there was nothing else on offer. But I think that was not their fault again, because the kitchen was not available then…’ (Participant 6) ‘I think the regimented three meals a day in hospital is a bit challenging for patients when they’ve got less appetite. And we would normally encourage a grazing approach to eating, to meet nutritional requirements. That’s a bit harder, especially some of the old ones that don’t have family to bring in snacks and things. And there isn’t that access on the wards and hospital as much.’ (Dietetic assistant)
	Social expectations in hospital and coping with difficult emotions	‘That’s another thing patients say all the time, they’ve got no appetite, because they’re just sat in bed all day. They’re not getting up, they’re not moving. They’re not doing anything. So, they don’t feel hungry because they’re not expending energy. And I suppose, some of them obviously can’t move about, like some of the orthopaedic patients with fractures. They physically can’t move about. But I have seen patients that could be walking about, that aren’t walking, and question why that’s the case, and whether nutrition would improve if they were up and about a bit more.’ (Dietetic assistant) ‘I know they say you should have a good breakfast. They feed like a king for breakfast, and then gradually reduce it during the day. But of course, I suppose in the hospital I was lying down for most of the day. Not walking about doing anything. But I just found that I preferred having a larger meal at midday. So, often I didn’t have a main course in the evening. I just enjoyed soup….’ (Participant 22) ‘…having a biscuit with a cup of tea, but a lot of the older people are too polite to ask. They don’t want to feel like they’re bothering people to ask for things. I think that’s quite a problem as well… when they do a tea round, offering a biscuit so the patients don’t have to ask. They don’t feel like they’re being a burden; that’s something I hear quite a lot.’ (Dietetic assistant)
	Staff attitudes and behaviours on a busy ward – beyond the written role	‘I mean the staff were very overworked. There never seems to be enough staff for everything going on. I think particularly because I was in the Geriatric ward, and there’s obviously a lot of problems for them aren’t there? When they have dementia patients they can take so much time for the staff because of their issues. It’s got to be very difficult to manage.’ (Participant 13) ‘I know I’m at the end of the age range where they think, ‘well, we’ll keep them alive, but not….’. that sounds terrible. But in hospital they just keep us alive. There was no progress forward; they were giving us medications, but there was no incentive to sort of improve, you know. I couldn’t get anyone to help me walk… Well, as I said, I sometimes asked for water in the night, and they’d say, ‘you must drink more’. But I’d ask for water, and it never came. The nurses went off and forgot about it. I did feel it was very poor…. It’s difficult cos the nurses have got to do what they’ve got to do, and you don’t want to be in their way.’ (Participant 6) ‘I think I wanted things cut up small. And if they were in big chunks it put me off. At home I cut things up very small. As far as I can remember I used to try and cut it up. And then I asked for a spoon to eat it. I feel as if I can’t chew big, anything hard. Skin of fruit for instance. I like grapes, but I can’t eat the skin. I’m always left with the skin in my mouth… Ah, maybe I didn’t ask. I don’t know. I would only eat things I could chew easily.’ (Participant 22) ‘I think the ward-based encouragement (is important). So, it’s the really basic ones like making sure that they’re sat out and they’re ready to eat a meal. That’s sometimes being missed on the wards. I know wards are working harder on it, but little things like that. And then just the encouragement to do that.’ (Dietetic assistant)
	Nutrition care pathways and staff practices influenced nutrition management	‘The trouble can be that they are physically incapable of feeding themselves. The other day I was one person looking after 2 patients; it was very hard to feed more than one patient at a time, so, therefore their food was getting colder; less appetising, and once it’s sat there for a while they’re less likely to want it. But you can’t physically feed. Unless you literally feed them a spoonful; walk over to the other one; feed them a spoonful, which I have done, but it’s silly. Staff numbers can be a barrier to nutrition. But that is why we let patients have visitors around mealtimes if they need help eating.’ (Nurse) ‘Already on the ward, we don’t take breaks at the same time as mealtimes. So, there’s more staff on the wards. The trouble is a lot of medication needs to be taken with food. So, you often do a medication round at lunchtime, that means you’re not available to help feed patients. You can’t do both in this situation.’ (Nurse) ‘There’s a drug bundle for anyone who comes in with a neck of femur (fracture). And as part of that they can be prescribed XXX (drink-based supplement). So, the doctors should tick that when someone comes in with a fractured neck of femur.’ (Dietician) ‘I think the nutrition, it gets overlooked by, more the medical team. They come in with fractures, or they come in with other medical needs. Nutrition isn’t the number one priority. And then it gets overlooked until it gets to a point where you can’t overlook it, at which point there’s so many things we could have intervened with earlier on to help.’ (Dietetic assistant) ‘The population group is so large that we don’t have enough staff to help every single malnourished older person in the hospital. And at the ward level, they don’t have the staffing or the resources always to provide as much assistance and encouragement at mealtimes as they would like to, or they should be offering.’ (Dietetic assistant)
Main theme	Quote examples	
Interaction between supplement texture, flavours, and presence of artificial ingredients influenced palatability and adherence	‘It (porridge) was glumpy. There were lumps in it, like the bran that was in it was still whole… The taste was okay if I could have got over the texture of it… There were still lumps and bits and pieces.’ (Participant 25) ‘I’ve had XXX (drink-based supplement) type of things before. And to be honest there’s nothing much there that I would say I liked. I find them a bit heavy; there was a fruity one that was okay, I could manage, but I’d say manage and not like. Because it was good for me; make myself eat it. But it’s the smell. Even the smell of them somehow puts me off.’ (Participant 6) ‘I thought it (porridge) was very good. I was so used to Sainsburys porridge it was nice to have one with a different flavour… I couldn’t fault that. I think the golden syrup one was the best.’ (Participant 8) ‘The drinks, I thought, were very nice. I mean, my favourite was Mocha, then Vanilla, then Strawberry. I liked them all, but Mocha was my favourite. And I quite looked forward to having those…’ (Participant 7) ‘I like the fact that it (porridge) is actual food, rather than a weird supplement drink, soup thing. Trouble is a lot of patients I talk to, it’s just so weird to have porridge plain like that without any milk on it; or any sugar; or anything. It’s just not how I would ever serve porridge… So, I liked the fact that it’s actual food. The patients liked that it was actual food.’ (Nurse) ‘Something as small as possible. Like trying to get that maximum nutrition in a smaller quantity of supplement as possible. Because a lot of time it’s the volume that they’re not managing.’ (Dietetic assistant) ‘It’s amazing how sick of something you get. And when the patients are being prescribed them (supplements) 3 times a day, I can really empathise with them when they go, ‘I just can’t drink another one of those!’.’ (Nurse)	

### Food traditions and lifelong preferences

Participants reported hospital food often did not meet their personal food preferences, reducing intake. Family traditions and eating cultures guided food intake and etiquette. For instance, most older people ate 3 small meals a day without snacks in-between meals, and they preferred whole foods to artificial supplements. Some participants grew up eating porridge in their families and preferred porridge as a traditional breakfast food rather than a snack. Staff reported porridge as an inappropriate supplement during the summer, where hot weather reduced calorie intake. Needing a specialist diet to manage disease symptoms (e.g., coeliac disease, diabetes) was perceived as a barrier to altering calorie and protein intake for some participants who were concerned about exacerbation of illness symptoms, especially if they consumed anything unfamiliar. Moreover, the association of supplements with end of life and illness deterred some participants from consumption.

### The interplay between age-related changes in physical ability and appetite and the consequences of illness and treatment influenced food intake

Reduced appetite due to medications and feeling ill impacted participant’s food intake, including adherence to supplements. Participants also noticed a slow decline in their appetite over time associated with their age and often reported preferring smaller portion sizes. Supplements with a smaller volume, a thinner texture, and drink-based, were easier to consume when participants had a reduced appetite. Weight loss and reduced strength motivated participants to increase their calorie intake. However, the presence of co-morbidities, poor dental health, constipation, impaired eyesight, fatigue, and pain changed some participants physical ability to prepare and eat food. Participants relied on food delivery services and family at home to assist with shopping and cooking and required assistance with choosing from the menu and cutting up food in hospital. Some participants required softer textures and had a slow eating pace, which resulted in cold unpalatable food. Supplements were often perceived as a good replacement for whole foods when a participant’s appetite was poor post-surgery and when eating was a struggle. Staff reported dementia as a significant risk for malnutrition, influencing appetite and ability to eat and becoming a barrier to understanding why they needed supplements.

### Hospital culture impacted food intake and supplement adherence

1. *Hospital food services influenced the availability and provision of palatable food*

The hospital provided a large variety of food options to meet patients’ food preferences and participants were pleasantly surprised by the quality of the food, especially given the large amounts of people that the hospital catered for. Only one participant disliked the food, and another found the food was served too cold, which reduced food intake. The hospital catered well for individuals requiring vegetarian and gluten-free options. However, participants reported a lack of ‘lighter’ food options, which they would have preferred post-surgery and with a poorer appetite. Staff explained the structure of three large meals a day conflicted with the dietetic teams’ advice to consume smaller more frequent meals throughout the day for older people with poor appetite. While biscuits were readily available as snacks, participants reported a lack of healthier snacks, such as fruit, and staff highlighted that improved access to hospital snacks was needed, offering a better variety of snacks and more often.

1. *Social expectations in hospital and coping with difficult emotions*

Participants reported feeling lonely on the wards and found the ward a depressing environment. Participants felt they had to stick to social hospital norms, including staying in bed and within their bay, which reduced feelings of well-being, increased sedentary behaviour, reduced physical activity and reduced appetite. Individuals who made social connections on the wards, had regular visitors, and were able to leave their bay, reported a stronger sense of well-being, which they believed enhanced their recovery. Families were pivotal to improving food intake, bringing in participants’ favourite food, helping with hospital food choices, and assisting at mealtimes. Patients who were not visited frequently and who were reluctant to ask for nutritional support from staff for fear of being a burden had reduced intake.

1. *Staff attitudes and behaviours on a busy ward – beyond the written role*

Nursing, health care support worker, and allied health care professional staff kindness and compassion improved patient recovery, but sometimes staff did not have the capacity to fulfil patient’s optimum nutritional needs. Patients with dementia disproportionately impacted on staff time that had an impact on other patients who reported feeling neglected. This influenced a negative view of staff attitudes to an ageing population where staff did ‘the basics to keep us alive’ but did not allow them to flourish. Most staff perceived ONS as an important part of malnutrition management. However, staff with negative perceptions of ONS influenced prescription and patient perspectives. Often the reasons for prescribing nutritional supplements and specialist nutritional advice were not readily available to patients; subsequently patients were unclear on the benefits of using supplements.

Basic help with eating and sourcing good nutrition was sometimes challenging in a busy ward environment. For instance, staff did not always have time to encourage patients to sit out of their beds to receive meals. Moreover, some patients reported staff frequently forgetting to give them fresh water, and staff were unavailable and/or unaware of participants that needed assistance with feeding, such as cutting food smaller to make chewing easier. When staff spent more time with patients to find out their personal needs, they were able to improve access to preferred foods and break hospital misconceptions. For example, advising on the importance of movement in hospital and if they were able, the opportunity for patients to leave the ward with a family member. Many patients did not want to bother busy staff, which sometimes compromised their optimum nutrition needs.

1. *Nutrition care pathways and established staff practices influenced nutrition management*

The dietetic team provided expert nutritional support. However, they were not available for every patient that needed them, and nutrition was often overlooked by the clinical care team or was perceived as a lower clinical priority. Nurses and health care assistants had key roles within a patients’ nutritional care, such as systematically monitoring weight and food intake. Nevertheless, wards were understaffed and there was poor capacity to implement optimal nutritional strategies. Staff outside the dietetic team required specialist nutritional training. However, high turnover of nurses and health care support workers due to shift work patterns typical on an acute ward created barriers to good nutritional support. Staff were encouraged to work at mealtimes to assist with feeding, though some nurses reported a clash with their duty to dispense medication at mealtimes. Participants recognised the value of family and volunteers, such as meal-time assistants to promote good nutrition. However, wards lacked implementation of such interventions.

Nutritional supplementation, including drink-based supplements and soups, were an established practice for malnourished patients in hospital. Supplements are given equal importance and were prescribed with usual medicines to facilitate administration. Moreover, clinical pathways, such as those for patients who have sustained a fractured neck of femur incorporate routine daily drink-based supplementation to reduce risk of malnutrition post-surgery. However, staff did not have capacity to monitor supplement adherence and offer alternative options for supplement refusals. Moreover, there were limited ONS options in hospital, in which a larger variety of ONS was needed to support a tailored approach. Drink-based supplements were perceived as convenient to provide compared to real food snacks and cheap to supply in hospital. Nursing staff wanted more input from the dietetic team to develop a nutritional care plan for patients on the wards and to review these plans before discharge, incorporating food fortification as well as ONS and arranging nutrition care follow-up in the community.

### Interaction between supplement texture, flavours, and presence of artificial ingredients influenced palatability and adherence

Five out of the nine participants interviewed enjoyed the taste of the drink-based supplement, 2 participants found the supplement too sweet, 1 participant said palatability would have improved with the drink served cold, and another disliked the smell and consistency of the supplement. Only 2 participants enjoyed the taste of the porridge, with golden syrup being the preferred flavour. The porridge was served hot, which was perceived as a facilitator to adherence. However, the thickness and texture of the porridge was frequently reported as a barrier to adherence, especially for individuals who found swallowing difficult. Having to prepare the powdered porridge created additional issues. For example, one participant reported the porridge was not mixed well enough and had powdered clumps, and another explained they preferred eating real oats rather than an artificial supplement. Some participants wanted to add other foods to the porridge to improve palatability including honey and Greek yoghurt.

Generally, staff reported that patients did not like the taste of supplements on offer in hospital, highlighting that they were often too sweet and that artificial ingredients used to increase shelf life impacted taste and palatability. Drink-based supplements were best served cold but there was not enough space in fridges on the wards. Staff liked the concept of porridge as an additional option as an ONS, as it looked and tasted less artificial. Additionally, their view was that supplements closest to the consistency and palatability to normal food improved patient adherence. Smaller supplement volumes and thinner, smoother consistencies were advocated in hospital to suit patients with poor appetite and difficulties eating. Having a better range of flavours and supplement types, including alternatives to milk-based options, was encouraged by staff, especially to combat supplement fatigue, i.e., boredom of consuming the same supplements.

## Discussion

This single-centre multi-method randomised controlled crossover study aimed to evaluate the adherence and palatability of a new porridge supplement compared to a drink-based supplement traditionally offered in hospital and to assess the acceptability of products with older people and healthcare professionals. Adherence was significantly higher with standard drink-based ONS (median 66.8% [IQR 26.29–75.37]; median 450.69 kcal [IQR 198.69–492.33]) compared to a new porridge supplement (median 26.31% [IQR 10.23–48.02]; median 141.41 kcal [IQR 63.13–249.32]) in hospitalised older people with medium-high risk of malnutrition. Overall, participants found the drink-based ONS more palatable than the porridge. Specifically, the texture of the porridge was significantly disliked compared to the drink-based control (Table 3). Four main themes were interpreted from interviews as influencing the acceptability of ONS and impacting participants’ food intake, including 1) food traditions and lifelong preferences, 2) the interplay between age-related changes in physical ability and appetite and the consequences of illness and treatment, 3) hospital culture, comprising hospital food services, social expectations in hospital, nutrition care pathways, and staff attitudes and behaviours on busy wards, and 4) interaction between supplement texture, flavours, and presence of artificial ingredients.

This study provided insight into the adherence and acceptability of a novel form of nutritional support in practice. Previous research has focused largely on drink-based ONS effectiveness and adherence, demonstrating average adherence rates of 67% in hospital.^(12)^ Of the few studies that have investigated other forms of supplementation in an older population, adherence rates have varied, such as 48.1% with homemade porridge and soup supplements,^(37)^ and 84.4% with an ice-cream-based supplement.^(21)^ Existing research demonstrated various reasons for poor adherence to ONS, including reduced palatability influenced by medications and dentures altering the mouthfeel of products.^(19)^ In the present study, aversion to the texture of the porridge likely contributed to poor adherence. Moreover, porridge (212.1 g) was a larger volume than the drink-based supplement (125 g) meaning a larger proportion of the porridge needed to be consumed to achieve daily energy and protein recommendations.^(8)^ Participants highlighted difficulties consuming supplements during hospitalisation caused by various factors such as reduced appetite due to illness, medications, and alterations in physical ability to eat. Therefore, our findings are consistent with recommendations of lower-volume energy-dense ONS in older people living with illness.^(12,38)^ Nevertheless, patients and staff indicated a desire for a better range of ONS in hospital, including supplements with fewer artificial ingredients. This presents challenges for industry to produce low-volume energy-dense products that do not compromise on taste.

Baseline daily energy intake (534.25 kcal) was lower (*p* = 0.004) compared to daily energy intake at porridge (790 kcal) and drink-based supplement (703.25 kcal) timepoints (excluding supplements), showing supplements did not replace normal dietary intake and indicating patient recovery over time. Total energy and protein intake across all timepoints in the current cohort was considered low even with the addition of supplements. Participants were not meeting nutritional requirements with a gap of 44–69% off daily energy requirements and 20–60% off protein requirements across study timepoints (i.e., baseline and supplementation). Interestingly, when comparing between porridge and drink-based supplement timepoints the gap to energy and protein requirements was similar despite lower adherence to the porridge supplement. Overall, the daily intake of supplements was not sufficient to improve participants’ energy and protein requirements.

Protein and energy intakes lower than requirements are associated with a range of negative health outcomes, including longer hospital stays, higher readmission rates, hospital-associated infections, frailty, and mortality.^(39,40)^ Consistent with our findings, decreased food intake in hospital is a prevalent and significant issue for older inpatients worldwide.^(41)^ For example, older adults (*n* = 81 age 81.5 ± 11.5 [mean ± SD] years) with malnutrition, or at risk of malnutrition in hospital in Italy, had a mean energy intake of 669.0 ± 573.9 (SD) kcal/day and mean protein intake of 30.7 ± 25.8 (SD) g/day during their first 5 days of hospital admission.^(41)^ This equated to 60% of patients who ingested ≤ 50% of their calculated energy and protein requirements.^(41)^

In line with existing literature, our interview findings highlighted various factors influencing older people’s food intake in hospital, including the impact of acute illness and medication on appetite, alterations in ability to eat (e.g., difficulties cutting food), oral problems (e.g., forgetting to bring dentures into hospital), catering limitations in hospital (e.g., restrictions on food serving times), reduced physical activity, and poor staff capacity to provide optimal nutritional support at meal times.^(31,41–43)^ Considering the multifactorial influences on patient appetite, participants’ gap to meeting energy and protein requirements, and the challenges associated with ONS consumption (e.g., dislike of textures and taste of artificial ingredients), our interview findings revealed the importance of additional nutritional strategies in hospital to manage and prevent malnutrition, including accessibility of snacks and the availability of family or volunteers to act as mealtime assistants. In a systematic review of 10 studies, alternative strategies, such as energy- and protein-based food fortification (e.g., protein-enriched bread or yoghurt) and snacks (e.g., cake, pastries, cheese sandwiches) were employed as an effective, acceptable, and cost-effective intervention to improve dietary intake among older people in hospital (546 patients), including a significant increase in energy (250–450 kcal per day) and protein intake (12–16 g per day).^(44)^ However, the feasibility of implementing such interventions is unclear. Indeed, our findings highlight the important impact of hospital food systems and ward culture on the availability and accessibility of nutritious food for patients. For instance, the hospital had limited snack choice and strict meal serving times, and ward staff often had limited time to deliver nutrition support, corroborated in previous literature showcasing poor nurse capacity to prioritise mealtimes above competing clinical demands.^(45)^ Future research is needed to assess the feasibility of adapting hospital food systems, including enrichment of hospital food menus and improving flexibility of mealtime services for older people with malnutrition.

Consistent with previous research,^(46)^ we showed that basic nutrition training was needed for a range of staff roles (e.g., health care assistants and nurses). Due to lower staff capacity and high staff turnover, the hospital adopted a ‘blanket’ approach to ONS prescription in certain clinical cohorts, such as automatically prescribing ONS to patients sustaining a neck-of-femur fracture, to overcome restricted access to the dietetic team and ensure patients at higher risk of malnutrition were receiving sufficient energy and protein pre- and post-operatively. However, staff reported drink-based ONS being discarded as they did not have the time to monitor adherence and offer alternative options to suit patient preferences. Therefore, to improve nutritional intervention effectiveness and sustainability, hospital departments need to consider person-centred strategies, catering for personal preferences, enabling eating pleasure, and being adaptable to different eating patterns.^(46)^ One such solution advocated by staff was assistance from family or volunteer mealtime assistants, which has a burgeoning evidence base that demonstrates improved dietary intake in hospitalised older people at risk of malnutrition.^(46,47)^ Enhanced staff training is also an important intervention, including educating on the importance of energy (calorie) and protein-first approach through food but complemented by the provision of ONS, ensuring good oral hygiene and that dentures are cleaned and fit well, comfortable positioning at mealtimes (e.g., encouraging patients to sit out of their bed), nursing assistance and supervision, and family involvement.^(47)^ Additionally, the concept of protected mealtimes should be prioritised where all health care professionals should refrain from clinical interventions, i.e., ward rounds, therapy.

In terms of good clinical practice, hospital policies and procedures should be reviewed and implemented in line with current clinical/research perspectives consistent with expert consensus. In the current study hospital screening policy advocated MUST to assess risk of malnutrition and guide patient nutrition plans. Evidence suggests MUST is a valid and reliable tool for screening malnutrition in hospital settings.^(22)^ However, there is increasing credibility worldwide for the diagnosis of malnutrition using the Global Leadership Initiative on Malnutrition (GLIM) criteria, working towards a common malnutrition ‘language’ to ensure malnutrition prevalence, interventions, and outcomes can be compared globally.^(48)^ Working towards the endorsement of malnutrition diagnostic criteria and management is needed for standard application in clinical settings. The ESPEN guideline on nutrition and hydration in geriatrics provides 82 recommendations to guide good clinical practice, including routine screening for malnutrition, ongoing systematic assessment of nutritional therapies, and individualising energy and protein requirements.^(8)^ In the current study optimal nutritional management was restricted by poor staff capacity and training, reduced availability of dietetic support, poor adherence to ONS, and inflexible hospital food systems. Interventions to improve food intake and ONS adherence should be individualised, comprehensive and part of a multimodal and multidisciplinary team approach,^(8)^ however wider hospital pressures and overarching health policies impact clinical practice and nutritional care, such as overcoming challenges related to staffing, equipment and funding.^(49)^

### Strengths and limitations

A strength of the study was objectively weighing the difference between prescribed and consumed ONS rather than relying on patient recall or estimating the proportion of ONS consumed. Moreover, the crossover design accounted for heterogeneous groups, such as differences in oral sensory perceptions and disease states. However, the small sample size, exclusion of individuals without capacity to consent, and single-centre design limit the generalisability of findings. Only 33% of patients approached participated in the study, which is consistent with recruitment challenges often experienced in an older population within health care research and points to the importance of reviewing and considering research processes to enhance inclusion of older people in clinical trials.^(51)^ Nevertheless, the qualitative interviews provided an in-depth exploration of factors influencing the acceptability and implementation of ONS products in hospital, meaningful insight into the barriers and facilitators of ONS consumption and showcased the complexity of appetite in acutely unwell older people and nutrition management in hospital systems.

There is a high prevalence of cognitive impairment secondary to dementia and delirium in hospitalised older people.^(52)^ These confer a higher malnutrition risk and present barriers to ONS adherence.^(53)^ Hence, future research investigating ONS adherence and palatability should include individuals without capacity to consent to ensure better representation of findings in practice. Furthermore, participants were mainly white British; hence, future research should improve recruitment strategies to include a wider diversity of older people. Consistent with previous research investigating adherence of innovative products,^(21)^ each product in the current study was tested over 2 days, which provided sufficient early insight into adherence and palatability allowing the development of products while saving resources. Nevertheless, further research examining adherence over a longer duration is needed to explore the possible effects of supplement fatigue, including re-measuring palatability after a longer consumption period for each product.

## Conclusion

Our findings highlight that adherence was significantly higher for standard drink-based ONS compared to a new porridge product. Older people at risk of malnutrition disliked the texture of the porridge and were unlikely to meet ONS energy and protein recommendations with a larger product volume given the impact of acute illness on reduced appetite compared to a smaller energy-dense drink-based supplement. Multifaceted influences on nutritional intake highlight the need for a multi-component approach to nutritional care in hospital, considering patient-centred care rather than relying on a ‘one-size fits all’ approach. To improve patient-centred nutritional care, participants requested a wider in-hospital range of ONS products with natural ingredients, enhanced training for staff in nutritional care, involvement from family and staff during mealtimes, and consideration of ‘real food’ options, such as food fortification and snacks. Future research is needed to develop in-hospital ONS options and to explore the feasibility of implementing wider nutritional care changes for hospitalised older people with malnutrition.

## Supporting information

10.1017/jns.2026.10108.sm001 Meredith et al. supplementary material 1 Meredith et al. supplementary material

10.1017/jns.2026.10108.sm002 Meredith et al. supplementary material 2 Meredith et al. supplementary material

10.1017/jns.2026.10108.sm003 Meredith et al. supplementary material 3 Meredith et al. supplementary material

## Supplementary material

The supplementary material for this article can be found at https://doi.org/10.1017/jns.2026.10108.

## References

[1] Salari N , Darvishi N , Bartina Y , et al. Global prevalence of malnutrition in older adults: a comprehensive systematic review and meta-analysis. Public Health Pract (Oxf). 2025;9:100583. 39885903 10.1016/j.puhip.2025.100583 PMC11780955

[2] Sobotka L. Basics in Clinical Nutrition. Galen; 2012.

[3] Cereda E , Pedrolli C , Klersy C , et al. Nutritional status in older persons according to healthcare setting: a systematic review and meta-analysis of prevalence data using MNA((R)). Clin Nutr. 2016;35(6):1282–1290. 27086194 10.1016/j.clnu.2016.03.008

[4] Leij-Halfwerk S , Verwijs MH , van Houdt S , et al. Prevalence of protein-energy malnutrition risk in European older adults in community, residential and hospital settings, according to 22 malnutrition screening tools validated for use in adults ≥65 years: a systematic review and meta-analysis. Maturitas. 2019;126:80–89. 31239123 10.1016/j.maturitas.2019.05.006

[5] Elia M , Stratton RJ. Calculating the cost of disease related malnutrition in the UK in 2007 (public expenditure only). In: Elia M and Russel CA , eds. Combating Malnutrition: Recommendations for Action A Report From the Advisory Group on Malnutrition Led by BAPEN. BAPEN; 2009:39–46.

[6] Norman K , Pichard C , Lochs H , Pirlich M . Prognostic impact of disease-related malnutrition. Clin Nutr. 2008;27(1):5–15. 18061312 10.1016/j.clnu.2007.10.007

[7] Rus GE , Porter J , Brunton A , et al. Nutrition interventions implemented in hospital to lower risk of sarcopenia in older adults: a systematic review of randomised controlled trials. Nutr Diet. 2020;77(1):90–102. 32022999 10.1111/1747-0080.12608 PMC7383582

[8] Volkert D , Beck AM , Cederholm T , et al. ESPEN guideline on clinical nutrition and hydration in geriatrics. Clin Nutr. 2019;38(1):10–47. 30005900 10.1016/j.clnu.2018.05.024

[9] Volkert D , Beck AM , Cederholm T , et al. Management of malnutrition in older patients-current approaches, evidence and open questions. J Clin Med. 2019;8(7):974. 10.3390/jcm8070974. 31277488 PMC6678789

[10] Milne AC , Potter J , Vivanti A , Avenell A . Protein and energy supplementation in elderly people at risk from malnutrition. Cochrane Database Syst Rev. 2009;2:CD003288. 10.1002/14651858.CD003288.pub3 PMC7144819 19370584

[11] Stratton RJ , Hebuterne X , Elia M. A systematic review and meta-analysis of the impact of oral nutritional supplements on hospital readmissions. Ageing Res Rev. 2013;12(4):884–897. 23891685 10.1016/j.arr.2013.07.002

[12] Hubbard GP , Elia M , Holdoway A , Stratton RJ . A systematic review of compliance to oral nutritional supplements. Clin Nutr. 2012;31(3):293–312. 22257636 10.1016/j.clnu.2011.11.020

[13] Jobse I , Liao Y , Bartram M , et al. Compliance of nursing home residents with a nutrient- and energy-dense oral nutritional supplement determines effects on nutritional status. J Nutr Health Aging. 2015;19(3):356–364. 25732222 10.1007/s12603-014-0544-y PMC12877517

[14] Gosney M. Are we wasting our money on food supplements in elder care wards? J Adv Nurs. 2003;43(3):275–280. 12859786 10.1046/j.1365-2648.2003.02710.x

[15] Hébuterne X , Frin G , Lefevere S , et al. Effectiveness and tolerance of an oral nutritional supplement highly concentrated in protein and energy in elderly subjects at risk of malnutrition. Nutrition Clinique et métabolisme. 2020;34:156–160.

[16] den Boer A , Boesveldt S , Lawlor JB. How sweetness intensity and thickness of an oral nutritional supplement affects intake and satiety. Food Qual Prefer. 2019;71:406–414.

[17] Thomas A , Van der Stelt AJ , Schlich P , Lawlor JB . Temporal drivers of liking for oral nutritional supplements for older adults throughout the day with monitoring of hunger and thirst status. Food Qual Prefer. 2018;70:40–48.

[18] Darmon P , Karsegard VL , Nardo P , et al. Oral nutritional supplements and taste preferences: 545 days of clinical testing in malnourished in-patients. Clin Nutr. 2008;27(4):660–665. 18625541 10.1016/j.clnu.2008.05.009

[19] Regan E , Feeney EL , Hutchings SC , et al. Food quality and preference, exploring how age, medication usage, and dentures effect the sensory perception and liking of oral nutritional supplements in older adults. 2021;92.

[20] Nieuwenhuizen WF , Weenen H , Rigby P , Hetherington MM . Older adults and patients in need of nutritional support: review of current treatment options and factors influencing nutritional intake. Clin Nutr. 2010;29(2):160–169. 19828215 10.1016/j.clnu.2009.09.003

[21] Taib A , Ong T , Mulvaney E , et al. Can an ice-cream based supplement help address malnutrition in orthogeriatric patients? J Nutr Gerontol Geriatr. 2021;40(4):280–289. 34635024 10.1080/21551197.2021.1984365

[22] Cortes R , Yanes AM , Capitan-Moyano L , et al. Evaluation of different screening tools for detection of malnutrition in hospitalised patients. J Clin Nurs. 2024;33(12):4759–4771. 38629350 10.1111/jocn.17170 PMC11579573

[23] Meredith SJ , Holt L , Varkonyi-Sepp J , et al. Frail2Fit study: it was feasible and acceptable for volunteers to deliver a remote health intervention to older adults with frailty. J Frailty Aging. 2025;14(6):100092. 41045456 10.1016/j.tjfa.2025.100092 PMC12519248

[24] Urbaniak GC , Plous PR. Research Randomizer. 2022; https://www.randomizer.org/

[25] Correa-Arruda WS , Vaez IDA , Aguilar-Nascimento JE , et al. Effects of overnight fasting on handgrip strength in inpatients. Einstein (Sao Paulo). 2019;17(1):eAO4418. 30652738 10.31744/einstein_journal/2019AO4418 PMC6333214

[26] Raiche M , Hebert R , Dubois MF. PRISMA-7: a case-finding tool to identify older adults with moderate to severe disabilities. Arch Gerontol Geriatr. 2008;47(1):9–18. 17723247 10.1016/j.archger.2007.06.004

[27] Ida S , Kaneko R , Murata K. SARC-F for screening of Sarcopenia among older adults: a meta-analysis of screening test accuracy. J Am Med Dir Assoc. 2018;19(8):685–689. 29778639 10.1016/j.jamda.2018.04.001

[28] Pilgrim AL , Baylis D , Jameson KA , et al. Measuring appetite with the simplified nutritional appetite questionnaire identifies hospitalised older people at risk of worse health outcomes. J Nutr Health Aging. 2016;20(1):3–7. 26728926 10.1007/s12603-016-0668-3 PMC4778266

[29] Wener MH , Daum PR , McQuillan GM. The influence of age, sex, and race on the upper reference limit of serum C-reactive protein concentration. J Rheumatol. 2000;27(10):2351–2359. 11036829

[30] Treacy M , Wong G , Odell M , Roberts N . Understanding the use of the National Early Warning Score 2 in acute care settings: a realist review protocol. BMJ Open. 2022;12(7):e062154. 10.1136/bmjopen-2022-062154 PMC9272106 35803636

[31] Pourhassan M , Cederholm T , Trampisch U , et al. Inflammation as a diagnostic criterion in the GLIM definition of malnutrition—what CRP-threshold relates to reduced food intake in older patients with acute disease? Eur J Clin Nutr. 2022;76(3):397–400. 34282291 10.1038/s41430-021-00977-4 PMC8907075

[32] National Institute for Health and Care Excellence. National early warning score systems that alert to deteriorating adult patients in hospital . 2020.

[33] Hoogendijk EO , van der Horst HE , Deeg DJH , et al. The identification of frail older adults in primary care: comparing the accuracy of five simple instruments. Age Ageing. 2013;42(2):262–265. 23108163 10.1093/ageing/afs163

[34] McGough C , Peacock N , Hackett C , et al. Taste preferences for oral nutrition supplements in patients before and after pelvic radiotherapy: a double-blind controlled study. Clin Nutr. 2006;25(6):906–912. 16774799 10.1016/j.clnu.2006.04.005

[35] Fiatarone Singh MA , Bernstein MA , Ryan AD , et al. The effect of oral nutritional supplements on habitual dietary quality and quantity in frail elders. J Nutr Health Aging. 2000;4(1):5–12. 10828934

[36] Braun V , Clarke V. Thematic Analysis: Practical Guide. Sage; 2022.

[37] Bunout B , Barrera B , de la Maza P , et al. Effects of nutritional supplementation and resistance training on muscle strength in free living elders. Results of one year follow. J Nutr Health Aging. 2004;8(2):68–75. 14978601

[38] Lombard K , van Steijn J , Schuur T , et al. Compliance of energy-dense, small volume oral nutritional supplements in the daily clinical practice on a geriatric ward--an observational study. J Nutr Health Aging. 2014;18(7):649–653. 25226102 10.1007/s12603-014-0496-2 PMC12880478

[39] Agarwal E , Ferguson M , Banks M , et al. Malnutrition and poor food intake are associated with prolonged hospital stay, frequent readmissions, and greater in-hospital mortality: results from the Nutrition Care Day Survey 2010. Clin Nutr. 2013;32(5):737–745. 23260602 10.1016/j.clnu.2012.11.021

[40] Thibault R , Makhlouf AM , Kossovsky MP , et al. Healthcare-associated infections are associated with insufficient dietary intake: an observational cross-sectional study. PLoS One. 2015;10(4):e0123695. 25923783 10.1371/journal.pone.0123695 PMC4414575

[41] Sanson G , Bertocchi L , Dal Bo E , et al. Identifying reliable predictors of protein-energy malnutrition in hospitalized frail older adults: a prospective longitudinal study. Int J Nurs Stud. 2018;82:40–48. 29579571 10.1016/j.ijnurstu.2018.03.007

[42] Cox NJ , Howson F , Ibrahim K , et al. Mood and physical activity are associated with appetite in hospitalised older men and women. Age Ageing. 2022;51(12):afac297. 10.1093/ageing/afac297. 36580556

[43] Cass AR , Charlton KE. Prevalence of hospital-acquired malnutrition and modifiable determinants of nutritional deterioration during inpatient admissions: a systematic review of the evidence. J Hum Nutr Diet. 2022;35(6):1043–1058. 35377487 10.1111/jhn.13009 PMC9790482

[44] Mills SR , Wilcox CR , Ibrahim K , et al. Can fortified foods and snacks increase the energy and protein intake of hospitalised older patients? A systematic review. J Hum Nutr Diet. 2018;31(3):379–389. 29322564 10.1111/jhn.12529

[45] Naughton C , Simon R , White TJ , et al. Mealtime and patient factors associated with meal completion in hospitalised older patients: An exploratory observation study. J Clin Nurs. 2021;30(19–20):2935–2947. 33945183 10.1111/jocn.15800

[46] Brunner S , Mayer H , Qin H , et al. Interventions to optimise nutrition in older people in hospitals and long-term care: umbrella review. Scand J Caring Sci. 2022;36(3):579–598. 34212419 10.1111/scs.13015 PMC9545538

[47] Zhang D , Tay LBG , Lim SF , et al. Improving nutrition care and diet intake for hospitalised older people at risk of malnutrition through a nurse-driven mealtime assistance bundle. Int J Older People Nurs. 2024;19(1):e12590. 37990475 10.1111/opn.12590

[48] Alves LF , de Jesus JDS , Britto VNM , et al. GLIM criteria to identify malnutrition in patients in hospital settings: a systematic review. J Parenter Enteral Nutri. 2023;47(6):702–709. 10.1002/jpen.2533 37314206

[49] Freedman S , Wolf R. The NHS Productivity Puzzle: Why Has Hospital Activity Not Increased in Line with Funding and Staffing?, Institute for Government. 2023.

[50] Vanderwee K , Clays E , Bocquaert I , et al. Malnutrition and nutritional care practices in hospital wards for older people. J Adv Nurs. 2011;67(4):736–746. 21143622 10.1111/j.1365-2648.2010.05531.x

[51] Goodwin VA , Low MSA , Quinn TJ , et al. Including older people in health and social care research: best practice recommendations based on the INCLUDE framework. Age Ageing. 2023;52(6):afad082. 10.1093/ageing/afad082. 37261448 PMC10234283

[52] Wu CR , Chang KM , Tranyor V , Chiu HY . Global incidence and prevalence of delirium and its risk factors in medically hospitalized older patients: a systematic review and meta-analysis. Int J Nurs Stud. 2025; 162:104959. 39602991 10.1016/j.ijnurstu.2024.104959

[53] Deeth S , Stevens S , Bell J , Mudge A . Nutrition care for older adults with delirium: a scoping review. J Clin Nurs. 2024;33(10):3886–3904. 38379358 10.1111/jocn.17069

