# Supplementary material for: A randomised controlled crossover study to assess adherence and palatability of a porridge supplement compared to a drink-based supplement in hospitalised older adults at risk of malnutrition
Source: J Nutr Sci. 2026 Jun 4;15:e40. doi: 10.1017/jns.2026.10108 (PMC13244357; doi:10.1017/jns.2026.10108)
Supplement: Meredith et al. supplementary material 2 — Meredith et al. supplementary material [file S2048679026101086sup002.docx]

**Supplement 2. Product Information**

| **Description** | Vital daily oats – Fortified Oatmeal, food first approach to malnutrition | High protein and energy, ready to drink, milkshake style supplement |
| --- | --- | --- |
| **Supplier** | Adams Clinical Nutrition Ltd. | Anonymous |
| **Weight** | 212.10g (with water) | 125g |
| **Energy kcal (per serving)** | 249 | 306 |
| **Protein (g)** | 16 | 18.3 |
| **Fat (g)** | 8.8 | 12 |
| **of which saturates (g)** | 2 | 1.08 |
| **Carbohydrate (g)** | 24.6 | 31.4 |
| **of which sugars (g)** | 7 | 17.1 |
| **Fibre (g)** | 5 | 0 |
| **Ingredients** | Golden syrup flavour: Organic gluten free oats, SOY Protein Isolate, Golden  flax seed, Full cream MILK powder, Sunflower seeds,  Organic millett, Organic cane sugar, Natural flavouring,  Vitamin premix*  *Vitamin K2 Menaquinone-7 (MK7), Vitamin E Natural  (D-Alpha-Tocopheryl Acetate), Vitamin D3  Cholecalciferol Powder, Beta Carotene (Blakeslea  trispora), Vitamin B5 Calcium Pantothenate, Vitamin A  Acetate (Retinol, Vitamin K1 (Phylloquinone /  Phytomenadione / Phytonadione), Vitamin B2 Riboflavin,  Vitamin B6 Pyridoxine HCl, Potassium Iodide, Vitamin  B12 Methylcobalamin (Mecobalamin). | Vanilla flavour: Cow’s **milk** proteins, water, maltodextrin, sucrose, vegetable oils (rapeseed oil, sunflower oil), magnesium hydrogen phosphate, emulsifier (**soy** lecithin), flavouring, choline chloride, potassium citrate, sodium L-ascorbate, dipotassium hydrogen phosphate, ferrous lactate, retinyl acetate, colour (curcumin), DL-α-tocopheryl acetate, copper gluconate, zinc sulphate, manganese sulphate, calcium D-pantothenate, thiamin hydrochloride, pyridoxine hydrochloride, nicotinamide, riboflavin, sodium fluoride, pteroylmonoglutamic acid, chromium chloride, potassium iodide, sodium molybdate, sodium selenite, D-biotin, phytomenadione, cholecalciferol, cyanocobalamin. Allergy Advice: For allergens, see ingredient in **bold**. |
