# Supplementary material for: A randomised controlled crossover study to assess adherence and palatability of a porridge supplement compared to a drink-based supplement in hospitalised older adults at risk of malnutrition
Source: J Nutr Sci. 2026 Jun 4;15:e40. doi: 10.1017/jns.2026.10108 (PMC13244357; doi:10.1017/jns.2026.10108)
Supplement: Meredith et al. supplementary material 3 — Meredith et al. supplementary material [file S2048679026101086sup003.docx]

**Interview Schedule – Patients**

1. **Explore general eating behaviours and food intake**
2. In general, what do you normally eat during the day?
3. Is there anything that puts you off from eating, or that you struggle with when eating, or getting food?

- Explore appetite
- Explore food preparation and cost
- Explore physical abilities (e.g., teeth, pain)

1. What kind of things encourage you to eat regularly, or makes eating easier?

- Explore support from others
- Taste preferences
- Food preparation

1. **Explore experiences of eating in hospital**
2. What are your views and experiences of the food you have eaten in the hospital?
3. Is there anything that puts you off from eating while you have been here in hospital, or that you struggle with when eating, or getting food?

- Explore appetite
- Explore food preparation and service
- Explore physical abilities (e.g., teeth, pain)

1. What kind of things encourage you to eat regularly, or makes eating easier in hospital?

- Explore support from staff
- Taste preferences

1. Did you need any help/support when eating your food? What was this support like?
2. **Explore experiences using ONS in hospital**
3. What did you think about the drinks (liquid-based ONS) you were given in-between your meals?

- What were the good things about the drink?
- Were there any bad things about the drink, or anything to improve?
- What did they taste like?
- Did you manage to drink both drinks over the day? Explore supplement fatigue/boredom

1. What did you think about the porridge (fortified ONS) you were given between your meals?

- What were the good things about the porridge?
- Were there any bad things about the porridge, or anything to improve?
- What did the porridge taste like? (explore palatability when hot and cold)
- Did you manage to eat both porridge supplements over the day? Explore supplement fatigue/boredom

1. What are your favourite snacks? Is there anything that you would prefer to eat in-between your meals in the hospital?
2. **Explore perceived eating/food improvements required in hospital**
3. What could improve your experience of eating food in the hospital?
4. What kind of food would you like to eat in hospital?
5. **Explore nutrition/diet transition from hospital to community**
6. When you leave hospital how confident are you that you can eat a healthy diet? Explore answer e.g., what makes you this confident?
7. Would you consider eating anything like the porridge or drinks in-between meals when you are at home? Why is this?
8. **Explore any other comments**
9. Do you have anything else to say about your diet/eating?
10. Do you have anything else to say about the porridge or drinks that you were given in-between your meals in hospital?

**Interview Schedule – Staff**

1. **Explore staff role in malnutrition management**
2. Could you explain what your job role is at the hospital?
3. Within your job role could you explain/describe the input you have regarding an older patient’s nutrition and eating?
4. If you come across a patient with malnutrition what is the process for treatment? In what ways does the hospital optimise nutrition for older patients with malnutrition?
5. **Explore general use of ONS in hospital**
6. In general, what is the prescription and management of ONS like in hospital for older malnourished patients?
7. What are your views about the use of ONS for older patients with malnutrition?
8. What are the benefits of using ONS?
9. What are your concerns with using ONS?
10. What are the barriers to using ONS in hospital with patients? In your view, what are the things that patients struggle with when using ONS?
11. How can these things be addressed? What factors facilitate the use of ONS in hospital with older patients?
12. **Explore specific ONS products used in the trial**
13. What do you think about the use of liquid-based ONS for malnourished patients in hospital?

- Benefits of the ONS?
- Disadvantages of the ONS, or anything to improve?

1. What do you think about the fortified porridge for malnourished older patients?

- Benefits of the porridge? Good things about the porridge?
- Disadvantages, or barriers when using the porridge?
- Would you use the porridge regularly with your patients? Why?

1. In your view, what are the best types of ONS to use in hospital? Why?
2. What needs to be changed to optimise patients’ nutrition in hospital?
3. **Explore perceptions of future improvements required for malnourished older adults nutritional care/support**
4. Do you feel you have sufficient support and resources to deliver optimal malnutrition management for older patients?
5. Does anything need to be changed to improve nutrition support for malnourished older adults in hospital?
6. Do you have anything else you would like to say about using ONS to manage malnutrition in hospital?
7. Do you have anything else you would like to add about the porridge or liquid-based ONS used in this study?
